# QuatJND: A Robust Quaternion JND Model for Color Image Watermarking

**DOI:** 10.3390/e24081051

**Published:** 2022-07-30

**Authors:** Wenbo Wan, Wenqing Li, Wenxiu Liu, Zihan Diao, Yantong Zhan

**Affiliations:** School of Information Science and Engineering, Shandong Normal University, Jinan 250358, China; wanwenbo@sdnu.edu.cn (W.W.); 2020020896@stu.sdnu.edu.cn (W.L.); 2020020900@stu.sdnu.edu.cn (W.L.); zdi230@uky.edu (Z.D.)

**Keywords:** quaternion JND, watermarking, contrast masking, colorfulness, robustness

## Abstract

Robust quantization watermarking with perceptual JND model has made a great success for image copyright protection. Generally, either restores each color channel separately or processes the vector representation from three color channels with the traditional monochromatic model. And it cannot make full use of the high correlation among RGB channels. In this paper, we proposed a robust quaternion JND Model for color image watermarking (QuatJND). In contrast to the existing perceptual JND models, the advantage of QuatJND is that it can integrate quaternion representation domain and colorfulness simultaneously, and QuatJND incorporates the pattern guided contrast masking effect in quaternion domain. On the other hand, in order to efficiently utilize the color information, we further develop a robust quantization watermarking framework using the color properties of the quaternion DCT coefficients in QuatJND. And the quantization steps of each quaternion DCT block in the scheme are optimal. Experimental results show that our method has a good performance in term of robustness with better visual quality.

## 1. Introduction

The protection of digital images is one of the urgent security issues that need to be solved nowadays, and digital image watermarking technology provides an effective solution. Digital image watermarking technology embeds watermarked information into multimedia information carriers without degrading the perceived quality but at the same time resists common attacks. The technology must satisfy robustness, imperceptibility and watermark capacity [1]. In past decades, digital image watermarking has been widely studied in grayscale images, whereas color images have received much less attention though they constitute most of the displayed multimedia content. Color information is also viewed as a significant feature in many fields of image processing. If correctly handled, color information will lead to more effective watermarking schemes, especially when achieving a good trade-off between imperceptibility and robustness [2]. Therefore, there is a considerable hot research topic for researchers to use the color information in digital image watermarking technology.

At present, most color image watermarking algorithms extract luminance information of color images or process only a single color channel, such as: (1) By transforming the color space model, the color image is transformed from RGB color space to YCbCr (or YUV) color space, and then the luminance component Y of the image is selected to embed the watermark; (2) According to the insensitivity of human vision system (HVS) to the change of blue component, the watermark is embedded by modifying the blue component value of color image [3]; (3) The three color channels of color images are processed separately, and watermark embedding also needs to be carried out on three color components respectively. Therefore, how to make better use of the correlation between the three channels of the color image is an issue that cannot be ignored.

In order to realize a better tradeoff between robustness and invisibility, the watermark strength can be achieved by the JND, which is the maximum distortion not perceived by HVS. The most well-known JND model is proposed by Watson et al. [4], the model consists of a sensitivity function, two masking components based on luminance and contrast masking. Lihong et al. [5] proposed robust algorithms which incorporate Watson’s model to compute the quantization steps, it has proved a significant improvement in robustness against the common attacks by the used perceptual model. In the past few years, the JND model has been the focus of research because of its excellent performance in the field of digital image analysis, such as Kim’s model [6], Zhang’s model [7], Wan’s model [8] and so on. And based on the development of JND modeling, some JND model-based watermarking algorithms are proposed [9,10,11]. In addition, visual saliency (VS) is also considered to facilitate JND metrics. However, these existing JND models restore each color channel separately or process the vector representation from three color channels with the traditional monochromatic model. And it cannot make full use of the high correlation among RGB channels. To account for this, a quaternion perceptual JND model is needed.

Quaternions, which have been increasingly used in color image processing in the past two decades, offer a solution to achieve this goal. They represent an image by encoding its three color channels on the imaginary parts of quaternion numbers. Compared with traditional color image processing technologies, the main advantage of such a representation is that a color image can be processed holistically as a vector field and can exploit the correlation between the three color components, so does the color image watermarking [12].

Recently, many algorithms have been proposed for color image watermarking based on Quaternion Discrete Fourier Transform (QDFT). Bas et al. [13] firstly proposed a non-blind color image watermarking algorithm in the QDFT domain by the method of quantization index modulation. But the algorithm has a low peak signal noise ratio (PSNR) and poor ability to resist attacks. Ma et al. [14] proposed a watermarking scheme for color images based on local quaternion Fourier spectral analysis (LQFSA). They introduced invariant feature transform (IFT) and geometric correction scheme to enhance the robustness to tackle geometric attacks. Jiang et al. [15] pointed out that, Bas et al. [13] didn’t consider the issue that the real part of the quaternion matrixes by inverse QDFT should be equal to zero and the problem could lead to a loss of watermark energy. They selected the real part of the QDFT coefficient matrixes to insert watermark and modified the coefficients of the real part symmetrically. Based on this constraint of symmetric distortion, Chen et al. [16] provided a Full 4-D quaternion discrete Fourier transform watermarking framework to illustrate the overall performance gain in terms of imperceptibility, capacity and robustness they can achieve compared to other quaternion Fourier transform based algorithms.

Furthermore, some other quaternion algorithms have been proposed, such as Quaternion Singular Value Decomposition (QSVD). In [17], a blind color image watermarking algorithm is proposed based on QSVD. The QSVD and rotation are employed to fulfill the process of watermarking and extracting watermark. Liu et al. [18] firstly performed QSVD to get U matrix and then the watermark was inserted into the optimally selected coefficients of the quaternion elements in the first column of the U matrix to enhance the invisibility. Recently, because the Discrete Cosine Transform (DCT) is compatible with the JPEG image compression standard, the watermarking algorithm in QDCT domain has received more considerable attention [19]. Therefore, it is meaningful to study how to introduce Quaternion Discrete Cosine Transform (QDCT) into watermarking algorithm.

In this paper, a robust quaternion JND model for color image watermarking (QuatJND) is proposed. And a novel and efficient robust quantization watermarking framework by exploiting quaternionic domain DCT based QuatJND model is proposed for color images. In our method, we embed the watermark into the QDCT domain by the method of spread transform dither modulation (STDM). At first, the colorfulness which is obtained in the QDCT domain is introduced as a new impact factor for QuatJND model. Furthermore, the QuatJND model is incorporated to derive the optimum quantization step for the embedding.

In summary, our main contributions are listed as follows:

(1) We proposed perceptual unit pure quaternion in the QDCT watermarking scheme. In this way, the proposed scheme can have the better performance.
(2) A quaternion perceptual JND model (QuatJND) is calculated in the QDCT domain.
(3) The color information and the pattern guided contrast masking effect in quaternion domain are considered for the QuatJND model.
(4) A logarithmic STDM watermarking scheme is proposed incorporate the QuatJND model. The proposed watermarking scheme can achieve a better performance with Peak Signal to Noise Ratio (PSNR) and Quaternion Structural Similarity Index (QSSIM).

The rest of this paper is organized as follows. Section 2 introduces the basic definitions that include quaternion and the QDCT of color images. Section 3 we provide QuatJND model which is used in the scheme and the colorfulness masking effect in quaternion DCT domain. Subsequently, we present the proposed watermarking scheme based on QDCT combines with QuatJND model. Experimental results and comparisons in Section 4 are provided to demonstrate the superior performance of the proposed scheme. Finally, we draw the conclusions in Section 5.

## 2. Quaternion DCT Definition

Quaternions were introduced by mathematician Hamilton in 1843 [20]. For easy reading, the main relevant abbreviations and symbols used in this paper is listed in Table 1.

Quaternion is the extension of real number and complex number, a quaternion has one real part and three imaginary parts given by

(1) q=a+bi+cj+dk

where a,b,c,d∈R, and i,j,k are three imaginary numbers which obey the following rules

(2) $$i^{2}=j^{2}=k^{2}=-1$$

(3) i·j=−j·i=k,k·i=−i·k=j,j·k=−k·j=i

If the real part a=0, *q* is called a pure quaternion.

Pei et al. [21] first applied quaternion to color image, as well proposed quaternion model of color image, which considered the three color components R, G, B as three imaginary parts of the quaternion. Let f(x,y) be an RGB image function with the quaternion representation (QR), then each pixel can be represented as a pure quaternion as

(4) $$f\left(x,y\right)=f_{R}\left(x,y\right)i+f_{G}\left(x,y\right)j+f_{B}\left(x,y\right)k$$

where $f_{R}\left(x,y\right)$, $f_{G}\left(x,y\right)$ and $f_{B}\left(x,y\right)$ are the pixel values of the R, G and B color components at position (x,y), respectively.

Because of the non-commutative multiplication rule for quaternions, the form of QDCT has two categories, left-handed form and right-handed form [19]. Without loss of generality, for QDCT, only the left-side one is considered in this paper, which satisfy the following equation

(5) $$C\left(p,s\right)=\alpha \left(p\right)\alpha \left(s\right)\sum_{x=0}^{M-1}\sum_{y=0}^{N-1}\mu \cdot f\left(x,y\right)\cdot N\left(p,s,x,y\right)$$

Corresponding to QDCT, the inverse Quaternion Discrete Cosine Transform (IQDCT) of f(x,y) is defined as

(6) $$f\left(x,y\right)=-\alpha \left(p\right)\alpha \left(s\right)\sum_{p=0}^{M-1}\sum_{s=0}^{N-1}\mu \cdot C\left(p,s\right)\cdot N\left(p,s,x,y\right)$$

where,

(7) $$N\left(p,s,x,y\right)=\cos\left[\frac{\pi (2x+1)p}{2M}\right]\cos\left[\frac{\pi (2y+1)s}{2N}\right]$$

and

(8) $$\alpha \left(p\right)=\left\{\begin{array}{cc} \; & \sqrt{\frac{1}{M}},p=0\\ \; & \sqrt{\frac{2}{M}},p\neq 0 \end{array}\right.$$

(9) $$\alpha \left(s\right)=\left\{\begin{array}{cc} \; & \sqrt{\frac{1}{N}},s=0\\ \; & \sqrt{\frac{2}{N}},s\neq 0 \end{array}\right.$$

and μ is a unit pure quaternion which meets the constraint that $\mu^{2}=-1$.

In order to reduce the complex computations and to make full use of the existing real-valued DCT codes, this subsection describes the relationship between QDCT and DCT. This relationship can provide not only an efficient computation approach for QDCT but also an approach to analyse the constraints for the watermark embedding.

Considering the general unit pure quaternion μ=ξi+ηj+γk, substituting Equation (4) into Equation (5), we have

(10) $$C\left(p,s\right)=\sum_{x=0}^{M-1}\sum_{y=0}^{N-1}\alpha (p)\alpha (s)\mu \cdot f\left(x,y\right)\cdot N\left(p,s,x,y\right)=C_{0}\left(p,s\right)+C_{1}\left(p,s\right)i+C_{2}\left(p,s\right)j+C_{3}\left(p,s\right)k$$

where,

(11) $$\begin{array}{cc} C_{0}\left(p,s\right) & =-[\xi DCT\left(f_{R}\left(x,y\right)\right)+\eta DCT\left(f_{G}\left(x,y\right)\right)+\gamma DCT\left(f_{B}\left(x,y\right)\right)]\\ C_{1}\left(p,s\right) & =\eta DCT\left(f_{B}\left(x,y\right)\right)-\gamma DCT\left(f_{G}\left(x,y\right)\right)\\ C_{2}\left(p,s\right) & =-\xi DCT\left(f_{B}\left(x,y\right)\right)+\gamma DCT\left(f_{R}\left(x,y\right)\right)\\ C_{3}\left(p,s\right) & =\xi DCT\left(f_{G}\left(x,y\right)\right)-\eta DCT\left(f_{R}\left(x,y\right)\right) \end{array}$$

$DCT(f_{R}\left(x,y\right)))$, $DCT(f_{G}\left(x,y\right)))$, $DCT(f_{B}\left(x,y\right))$, are respectively the conventional DCT matrix of the red, green and blue channels, and DCT(·) is the conventional discrete cosine transform.

Similarly, applying IQDCT, we get the reconstructed image

(12) $$\begin{array}{c} \bar{f}\left(x,y\right)=-\sum_{p=0}^{M-1}\sum_{s=0}^{N-1}\alpha (p)\alpha (s)\mu \cdot C\left(p,s\right)\cdot N\left(p,s,x,y\right)=\bar{f_{0}}\left(x,y\right)+\bar{f_{1}}\left(x,y\right)i+\bar{f_{2}}\left(x,y\right)j+\bar{f_{3}}\left(x,y\right)k \end{array}$$

where,

(13) $$\begin{array}{cc} \bar{f_{0}}\left(x,y\right) & =[\xi IDCT\left(C_{1}\left(p,s\right)\right)+\eta IDCT\left(C_{2}\left(p,s\right)\right)+\gamma IDCT\left(C_{3}\left(p,s\right)\right)]\\ \bar{f_{1}}\left(x,y\right) & =-[\xi IDCT\left(C_{0}\left(p,s\right)\right)+\eta IDCT\left(C_{3}\left(p,s\right)\right)-\gamma IDCT\left(C_{2}\left(p,s\right)\right)]\\ \bar{f_{2}}\left(x,y\right) & =-[-\xi IDCT\left(C_{3}\left(p,s\right)\right)+\eta IDCT\left(C_{0}\left(p,s\right)\right)+\gamma IDCT\left(C_{1}\left(p,s\right)\right)]\\ \bar{f_{3}}\left(x,y\right) & =-[\xi IDCT\left(C_{2}\left(p,s\right)\right)-\eta IDCT\left(C_{1}\left(p,s\right)\right)+\gamma IDCT\left(C_{0}\left(p,s\right)\right)] \end{array}$$

Here, IDCT(·) is the conventional inverse discrete Cosine transform.

For the color image signal, it can be drawn from Equation (12) that IQDCT must be a pure quaternion matrix after modifying some QDCT coefficients to insert watermark. Otherwise, taking only the three imaginary parts of this quaternion matrix to get the watermarked image will discard non-null real part data and result in a loss of watermark energy. Based on the above relationships Equations (10) and (12) and depending on the pure unit quaternion considered, one can identify the constraint to respect when modifying QDCT coefficients so as to avoid watermark energy loss. After the watermark embedding process, $\bar{f}$ should be a pure quaternion, or more clearly

(14) $$\bar{f_{0}}=0$$

where 0 is a zero matrix.

For the IQDCT coefficients matrix, we can obtain the real part from Equation (13) as

(15) $$\begin{array}{c} \bar{f_{0}}\left(x,y\right)=\left[\xi IDCT\left(C_{1}\left(p,s\right)\right)+\eta IDCT\left(C_{2}\left(p,s\right)\right)+\gamma IDCT\left(C_{3}\left(p,s\right)\right)\right] \end{array}$$

In order to respect the constraint Equation (14), as we can see from Equation (15), $\bar{f_{0}}$ is not related to one component $C_{0}\left(p,s\right)$. So, if we modify $C_{0}\left(p,s\right)$ to insert watermark, the precondition $\bar{f_{0}}=0$ is satisfied.

## 3. Proposed Method

### 3.1. Perceptual Unit Pure Quaternion

To avoid watermark energy loss, the real part $C_{0}\left(p,s\right)$ after QDCT is selected to embed watermarking. It can be seen form Equation (16), for different unit pure quaternion, the $C_{0}\left(p,s\right)$ part transformation coefficients are different, and the schemes of modifying coefficient embedding watermark are also different. Hence, the combination of unit pure quaternion and its weight will affect the performance of watermarking algorithm.

(16) $$\begin{array}{c} C_{0}\left(p,s\right)=-\left[\xi DCT\left(f_{R}\left(x,y\right)\right)+\eta DCT\left(f_{G}\left(x,y\right)\right)+\gamma DCT\left(f_{B}\left(x,y\right)\right)\right] \end{array}$$

$DCT(f_{R}\left(x,y\right)))$, $DCT(f_{G}\left(x,y\right)))$, $DCT(f_{B}\left(x,y\right))$ are respectively the conventional DCT matrix of the red, green and blue channels. Therefore, $C_{0}\left(p,s\right)$ part can be deemed a weighted aggregate of each component of the color such as R, G, and B. Although we embed watermark information into $C_{0}\left(p,s\right)$ part which changes the distribution of the values of $C_{0}\left(p,s\right)$, for the whole image in spatial domain, the differences can spread to the R, G, B three color components.

During the QDCT transformation, a unit pure quaternion $\mu =\left(i+j+k\right)/\sqrt{3}$ is the most commonly used where $\xi =\eta =\gamma =1/\sqrt{3}$. The unit pure quaternion will cause the same amount of change in the three color components of R, G, and B. However, due to the color sensitivity of the human eye to R, G and B is different, this change will make the invisibility of watermarking method is poor. In order to improve the invisibility of the watermarking scheme, we proposed the perceptual unit pure quaternion.

In the process of exploring the weight of ξ, η, and γ, we find that Zhu et al. [22] pointed out the RGB input signal can be converted into the YCbCr signal to remove the redundancies across three color channels and to offer good experimental results. The luminance component Y can be represented use the R, G, B three color components and the weight of R, G and B is 0.299, 0.587 and 0.114, respectively. And, some color image watermarking algorithms such as in YCbCr (or YUV) space [23,24], they modified the luminance component to inject watermark, and the experimental results showed good invisibility.

Therefore, to obtain well imperceptibility of the watermarked model, the unit pure quaternion and its weight according to relative relationship between the color channel R, G, and B is 0.299, 0.587 and 0.114, respectively. And the unit pure quaternion which should meet the constraint that $\mu^{2}=-1$. Then the perceptual unit pure quaternion is

(17) $$\mu =\xi^{*}i+\eta^{*}j+\gamma^{*}k$$

and,

(18) $$\begin{array}{cc} \mu^{2}= & \left(\xi^{*}i+\eta^{*}j+\gamma^{*}k\right)\left(\xi^{*}i+\eta^{*}j+\gamma^{*}k\right)\\ \;= & -{\left(\xi^{*}\right)}^{2}+\xi^{*}\eta^{*}k-\xi^{*}\gamma^{*}j-\xi^{*}\eta^{*}k-{\left(\eta^{*}\right)}^{2}+\eta^{*}\gamma^{*}i+\xi^{*}\gamma^{*}j-\eta^{*}\gamma^{*}i-{\left(\gamma^{*}\right)}^{2}\\ \;= & -{\left(\xi^{*}\right)}^{2}-{\left(\eta^{*}\right)}^{2}-{\left(\gamma^{*}\right)}^{2}\\ \;= & -1 \end{array}$$

where, the perceptual unit pure quaternion μ and its weight $\xi^{*}$, $\eta^{*}$, and $\gamma^{*}$ is 0.299:0.587:0.114, substituting the relative relationship into Equation (18), and we can obtain the $\xi^{*}\;=\;0.4472$, $\eta^{*}\;=\;0.8780$, and $\gamma^{*}\;=\;0.1705$, respectively. The experimental results are provided in Section 4.3.1 show that the perceptual pure unit quaternion μ has the better performance.

### 3.2. Proposed Quaternionic JND Model

For an image, a high-precision perceptual JND profile is usually perceived various changes which includes the spatial contrast sensitivity function (CSF), luminance adaptation (LA) effect and the contrast masking (CM) effect. In fact, the color sensitivity needs to be concerned for a perceptual JND profile in color images. The JND in the QDCT domain is typically expressed as a product of a base threshold and some modulation factor. In this paper, the real part $C_{0}\left(p,s\right)$ after QDCT is selected to embed watermarking. To obtain the JND threshold of the modified coefficients in $C_{0}\left(p,s\right)$, in this section, a novel contrast masking effects considering colorfulness is introduced:

(19) $$JND\left(t,m,n\right)=\tau \cdot N\cdot J_{q\_base}\cdot M_{q\_CM}\cdot M_{q\_LA}\cdot M_{q\_COL}$$

where the parameter *t* is the index of a QDCT block, and (m,n) is the position of the QDCT block coefficients. τ is to account for the summation effect of individual JND thresholds over a spatial neighborhood for the visual system and is set to 0.14. *N* is the dimension of QDCT (8 in this case). $J_{q\_base}$ is the base CSF threshold, $M_{q\_LA}$ is the LA effect and $M_{q\_CM}$ is the CM effect [8,9,25,26]. And $M_{q\_COL}$ is an important factor to reflect the colorfulness.

#### 3.2.1. Spatial CSF in Quaternion Domain

$J_{q\_base}$ is the quaternion domain JND value for the component $C_{0}\left(p,s\right)$ generated by spatial CSF on a uniform background image [6] and can be given by considering the oblique effect in QDCT domain as

(20) $$\begin{array}{cc} J_{q\_base}= & \left(J_{q\_d}\left(\omega_{m,n}\right)-J_{q\_v}\left(\omega_{m,n}\right)\right)\cdot \sin{\left(\varphi_{m,n}\right)}^{2}+J_{q\_v}\left(\omega_{m,n}\right) \end{array}$$

where $J_{q\_d}\left(\omega_{m,n}\right)$ and $J_{q\_v}\left(\omega_{m,n}\right)$ is formulated by QDCT coefficients

(21) $$\left\{\begin{array}{c} J_{q\_d}\left(\omega_{m,n}\right)=0.0293\cdot {\omega_{m,n}}^{2}+\left(-0.1382\right)\cdot \omega_{m,n}+1.75\\ J_{q\_v}\left(\omega_{m,n}\right)=0.0238\cdot {\omega_{m,n}}^{2}+\left(-0.1771\right)\cdot \omega_{m,n}+1.75 \end{array}\right.$$

where $\omega_{m,n}$ is cycle per degree (cpd) for the (m,n)-th QDCT coefficient and is given by

(22) $$\omega_{m,n}=\sqrt{m^{2}+n^{2}}/\left(2N\theta \right)$$

and,

(23) $$\theta =\tan^{-1}\left[1/2\cdot R_{VH}\cdot H\right]$$

where θ indicates the horizontal/vertical length of a pixel in degrees of visual angle, $R_{VH}$ is the ratio of the viewing distance to the screen height, and *H* is the number of pixels in the screen height. $\varphi_{m,n}$ stands for the direction angle of the corresponding QDCT component, which is expressed as

(24) $$\varphi_{m,n}=\sin^{-1}\left(2\cdot \omega_{m,0}\cdot \omega_{0,n}/{\omega_{m,n}}^{2}\right)$$

#### 3.2.2. Luminance Adaptation in Quaternion Domain

An luminance adaptation factor $M_{q\_LA}$ that employed both the cycles per degree (cpd) $\omega_{m,n}$ for spatial frequencies and the average intensity value of the block $\mu_{la}$ can be formulated as,

(25) $$M_{q\_LA}=\left\{\begin{array}{c} 1+\left(M_{0,1}-1\right){\left|\frac{\mu_{la}-0.3}{0.2}\right|}^{0.8},\mu_{la}\leq 0.3\\ 1+\left(M_{0,9}-1\right){\left|\frac{\mu_{la}-0.3}{0.6}\right|}^{0.6},\mu_{la}>0.3 \end{array}\right.$$

where the $M_{0,1}$, $M_{0,9}$ are empirically set as

(26) $$\left\{\begin{array}{c} M_{0,1}=2.468\times 10^{-4}{\omega_{m,n}}^{2}+4.466\times 10^{-3}\omega_{m,n}+1.14\\ M_{0,9}=1.230\times 10^{-4}{\omega_{m,n}}^{2}+1.433\times 10^{-3}\omega_{m,n}+1.34 \end{array}\right.$$

where $\omega_{m,n}$ is expressed as in Equation (22) and the average intensity value of the t-block $\mu_{la}$ can be expressed as

(27) $$\mu_{la}=C_{0}\left(0,0\right)\cdot \frac{C}{E_{d}}$$

where $C_{0}\left(0,0\right)$ is the QDCT coefficient at position (0,0) of the t-th $C_{0}$ block called Q-DC coefficient (Quaternion DC coefficient). $E_{d}$ denotes the maximum directional energy of image block in Equation (28), *C* is a fixed constant and is approximately equal to $E_{d}$ to ensure the invariance and stability of $\mu_{la}$. Therefore, the proposed formula can resist the fixed gain attack as it will vary linearly with the amplitude changes.

(28) $$E_{d}=\max\left(\left|C_{0}\left(0,1\right)\right|,\left|C_{0}\left(1,0\right)\right|,\left|C_{0}\left(1,1\right)\right|\right)$$

where $C_{0}\left(0,1\right)$, $C_{0}\left(1,0\right)$ and $C_{0}\left(1,1\right)$ are the QDCT coefficients at position (0,1), (1,0) and (1,1) of the t-th $C_{0}$ block called Q-AC coefficient (Quaternion AC coefficient). Similar to DCT transformation [27], the Q-AC coefficients obtained after QDCT transformation can reflect the image block direction energy. In our work, we select $C_{0}\left(0,1\right)$, $C_{0}\left(1,0\right)$, $C_{0}\left(1,1\right)$ to reflect the directional energy of the block in the horizontal, vertical and diagonal direction, respectively.

#### 3.2.3. Pattern Guided Contrast Masking in Quaternion Domain

$M_{q\_CM}$ is modeled for boosting the $J_{q\_base}$ based on local spatial texture complexity (e.g., smoothness, edge or texture), which is given by

(29) $$\begin{array}{ccc} \; & M_{q\_CM}=\; & \left\{\begin{array}{c} 1+\left(g_{1}\left(\omega_{m,n}\right)-1\right)\cdot \left(\frac{\mu_{cm}}{0.15}\right),0\leq \mu_{cm}<0.15\\ g_{1}\left(\omega_{m,n}\right),0.15\leq \mu_{cm}<0.2\\ g_{1}\left(\omega_{m,n}\right)+\left(g_{2}\left(\omega_{m,n}\right)-g_{1}\left(\omega_{m,n}\right)\right)\cdot \left(\frac{\mu_{cm}-0.2}{0.1}\right),\mathrm{others} \end{array}\right. \end{array}$$

where the $g(\omega_{m,n})$ is modeled in a gamma pdf form and expressed as

(30) $$g_{l}\left(\omega_{m,n}\right)=\left(\left(\frac{\beta^{\alpha_{cm}}}{\Gamma (\omega )}\right)\omega^{\alpha_{cm}-1e^{-\beta_{cm}\omega }}\right)\cdot \gamma_{cm}+\delta_{m}$$

(31) $$\left\{\begin{array}{c} g_{l=1}\left(\omega_{m,n}\right):\alpha_{cm}=3.4,\beta^{\alpha_{cm}}=2,\gamma_{cm}=8.0,\delta_{m}=1.42\\ g_{l=2}\left(\omega_{m,n}\right):\alpha_{cm}=3.4,\beta^{\alpha_{cm}}=2,\gamma_{cm}=12.4,\delta_{m}=2.83 \end{array}\right.$$

where, $\mu_{cm}$ represents the contrast masking effect of t-th QDCT block. In this paper, both pattern complexity and luminance contrast are considered to construct the contrast masking effect. And the contrast masking effect $\mu_{cm}$ is defined as

(32) $$\mu_{cm}=f(C_{p})\cdot \mu \left(C_{l}\right)$$

where, $C_{p}$ is the pattern complexity and $C_{l}$ is the luminance contrast of t-th QDCT block, respectively.

The pattern complexity measurement of the block proposed by Wan et al. [9] is the ratio of the maximum directional energy and the DC coefficient of each 8×8 block, which can measure energy in different directions while keeping the measurement of pattern complexity insensitive to the changes caused by the watermarking process. However, this method ignores the relationship between the directional energy of a DCT block and its neighboring DCT blocks. Therefore, we propose a new pattern complexity representation that combines the directional energy within a QDCT block and the directional energy of its neighboring QDCT blocks. This method is more effective in representing the complexity relationship of image patterns.

Firstly, we choose a neighborhood of size 3×3 for each 8×8 QDCT block. If the directional location of the maximum directional energy of its neighboring block is the same as this QDCT block, then the neighboring block is marked. We choose the ratio of the number of marked neighborhood blocks to all neighborhood blocks of this QDCT block as the pattern complexity $C_{p}$.

Therefore, the pattern complexity $C_{p}$ of the image block is represented by

(33) $$C_{p}=\frac{\sum_{i=1}^{n}D_{i}}{n}$$

where, $D_{i}$ represents the correlation between the image block and its neighbor in Equation (34), and *n* is the number of neighborhoods of t-th QDCT block.

(34) $$D_{i}=\left\{\begin{array}{c} 1,location\left(E_{d}\right)=location\left(E_{d,i}\right)\\ 0,else \end{array}\right.$$

where, $E_{d}$ is the maximum directional energy of t-th QDCT block and $E_{d,i}\left(i=1,2,\ldots ,n\right)$ are the maximum directional energy of neighboring blocks of the t-th QDCT block.

Since the pattern complexity of the irregular regions in the image is stronger, the diminishing effect of $C_{p}$ follows the non-linear transducer as

(35) $$f\left(C_{p}\right)=1-0.2\cdot {C_{p}}^{0.7}$$

The luminance contrast $C_{l}$ can be obtained from Q-AC coefficients $C_{0}\left(0,1\right)$, $C_{0}\left(1,0\right)$ and $C_{0}\left(1,1\right)$

(36) $$C_{l}=∐\left(\sqrt{{C_{0}\left(0,1\right)}^{2}+{C_{0}\left(1,0\right)}^{2}+{C_{0}\left(1,1\right)}^{2}}\right)$$

where, ∐(·) is normalization operation. Following logarithmic form, the increasing effect of $C_{l}$ can be represented as

(37) $$\mu \left(C_{l}\right)=\ln\left(1+0.47\cdot C_{l}\right)$$

**Figure 1:**
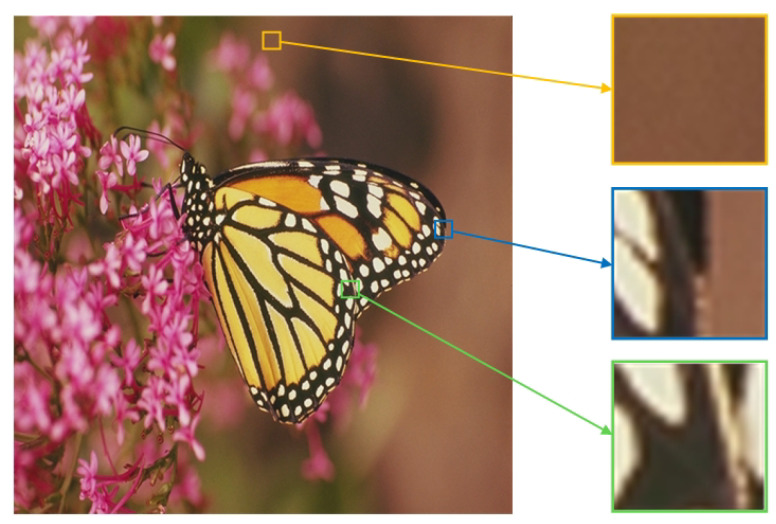
The $\mu_{cm}$ of three types of image blocks. The $\mu_{cm}$ of yellow image block is 0.0035; the $\mu_{cm}$ of blue image block is 0.1886; the $\mu_{cm}$ of green image block is 0.2481.

Figure 1 shows the $\mu_{cm}$ of three types of image blocks, such as smoothness, edge and texture. The yellow image block is smooth, and its $\mu_{cm}$ is less than 0.15. The blue image block is an edge block whose $\mu_{cm}$ is greater than 0.15 and less than 0.2. An image block with its $\mu_{cm}$ greater than 0.2 is a texture block, such as the green image block in Figure 1.

#### 3.2.4. Colorfulness Masking in Quaternion Domain

In this part, we proposed a new masking function that consider the colorfulness masking effect from $C_{1}$, $C_{2}$ and $C_{3}$ parts. For the color images, when human eyes observe different colors, the interaction between different colors will interfere with the judgment of color. Colorfulness is the attribute of chrominance information humans perceive. Hasler and Susstrunk [28] have shown that colorfulness can be represented effectively with combinations of image statistics ( the variance and mean values). And Panetta et al. [29] pointed out, just as the human visual system (HVS), human eyes capture color information in the opponent color spaces such as red-green (R-G) and yellow-blue (Y-B) color space. In a word, the colorfulness can be formulated by using image statistics in opponent color spaces.

In this paper, we select $C_{1}$, $C_{2}$ and $C_{3}$ parts after QDCT to calculate the image block’s colorfulness. In QDCT domain, we are first transformed into the opponent red-green and yellow-blue color space can be expressed as follows:

(38) $$\begin{array}{cc} \; & K_{1_{\left(R-G\right)}}=-C_{3}\\ \; & K_{2_{\left(Y-B\right)}}=C_{2}-C_{1} \end{array}$$

Then, for a QDCT block (8×8), the image colorfulness $Q_{c}$ is defined as

(39) $$Q_{c}=\left(\sqrt{\sigma_{K_{1}}^{2}+\sigma_{K_{2}}^{2}}+0.3\sqrt{\mu_{K_{1}}^{2}+\mu_{K_{2}}^{2}}\right)/85.59$$

where, $\sigma_{K_{1}}^{2}$, $\sigma_{K_{2}}^{2}$, $\mu_{K_{1}}^{}$ and $\mu_{K_{2}}^{}$ represent the variance and mean values along these two opponent color axes and can be expressed by the coefficients of QDCT block

(40) $$\mu_{K_{1}}^{}=\frac{1}{N}\sum_{p=1}^{N}K_{1_{p}}$$

(41) $$\sigma_{K_{1}}^{2}=\frac{1}{N}\sum_{p=1}^{N}\left(K_{1_{p}}^{2}-\mu_{K_{1}}^{2}\right)$$

**Figure 2:**
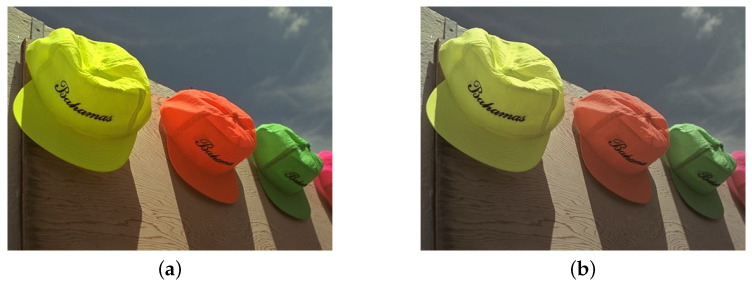
Image comparisons of colorfulness metrics. (**a**) Reference image with colorfulness is 0.9462; (**b**) Chrominance distortion image with colorfulness is 0.4563.

Figure 2 shows the comparisons of colorfulness metrics. Figure 2a,b are from TID2008 database [30]. The colorfulness of Figure 2a,b is 0.9462 and 0.4563, respectively. The results indicate the colorfulness metrics have a good correlation with human color perception. Inspired by this, a factor obtained from colorfulness is used to make JND a better match for human beings. The colorfulness masking factor $M_{q\_COL}$ is defined as

(42) $$M_{q\_COL}=1+\left(∐Q_{C}-0.3\right)\cdot 0.28$$

where, ∐(·) is normalization operation.

### 3.3. QuatJND-Based Watermarking

In this section, the flowchart of the proposed watermarking scheme based QuatJND model is briefly introduced.

#### 3.3.1. Adaptive Quantization Step

In this paper, some of the QDCT coefficients denoted as the host vector *X*, the maximum imperceptible change in the random direction of *v* can be given as $X^{T}v$. To ensure the independence between the quantization compensation and the original signal in the watermarking process, the host vector is transformed into logarithmic domain firstly.

(43) $$Y=F\left(X^{T}v\right)=\frac{\ln(1+z\frac{X^{T}v}{E_{d}})}{\ln(1+z)}$$

where, *v* is the random projection vector, $E_{d}$ is the image block direction maximum energy in Equation (28), which is to resist the linear variation. And *z* is used as a secret key.

In this arrangement, the transformed vector *Y* is quantized into $Y_{w}$ regarding the watermark bit as

(44) $$\begin{array}{cc} \; & Y_{w}=Q\left(Y,\Delta ,w,d_{m}\right)=\Delta \cdot round\left(\frac{Y+d_{m}}{\Delta }\right)-d_{m},w\in \left\{0,1\right\} \end{array}$$

where, $d_{m}$ is the dither signal corresponding to the message bit w and the proposed JND model can be used as a slack S to calculate the adaptive quantization step Δ

(45) $$\Delta =\ln\left(1+\frac{2S^{T}v}{E_{d}}\right)/\ln\left(1+z\right)$$

Thus, when the image is scaled by a fixed gain, the coefficients to be watermarked and the estimated quantization step Δ can ensure stability.

#### 3.3.2. Watermark Embedding Procedure

**Figure 3:**
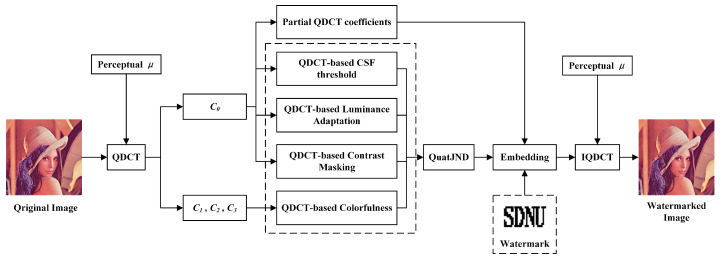
The flowchart of the proposed watermark embedding scheme.

The proposed watermarking scheme includes two parts, embedding and extraction procedure. Figure 3 illustrates the embedding steps of the watermarking scheme. Here, taking Lena image as an example, the procedures of the watermark embedding are shown as follows:

Step 1: For an original image, it is first divided into non-overlapped blocks of 8×8 size, and each block is converted to the quaternion representation by Equation (4).

Step 2: Apply QDCT which used the perceptual unit pure quaternion μ to each block, and the QDCT spectrum coefficients are obtained by Equation (11).

Step 3: Estimate the QuatJND factors including the spatial CSF effect, luminance adaptation and contrast masking in $C_{0}$ quaternion domain by Equations (20), (25) and (29), respectively.

Step 4: Extract colorfulness feature from $C_{1}$, $C_{2}$ and $C_{3}$ by Equation (39). Quantize and calculate the colorfulness masking of each 8×8 block for QuatJND profile by Equation (42).

Step 5: Final QuatJND value of each block combined with colorfulness masking is determined by Equation (19). The proposed QuatJND value can be served as the perceptual redundancy vector *S*.

Step 6: The $C_{0}$ coefficients from the fourth to tenth except the fifth after zigzag scan are selected to form a host vector *X*. The host vector *X* and the perceptual redundancy vector *S* are used to obtain the transformed vector *Y* and the adaptive quantization step Δ.

Step 7: One bit of the watermark message *w* after Arnold transformation is embedded into the transformed vector *Y* as followed:

(46) $$Y_{w}=Q\left(Y,\Delta ,w,d_{m}\right)$$

Step 8: Transform the modulated coefficients $Y_{w}$ back to form the watermarked image $X_{w}$.

Step 9: Finally, the inverse QDCT on each block is performed, and then the watermarked image is obtained.

#### 3.3.3. Watermark Extracting Procedure

**Figure 4:**
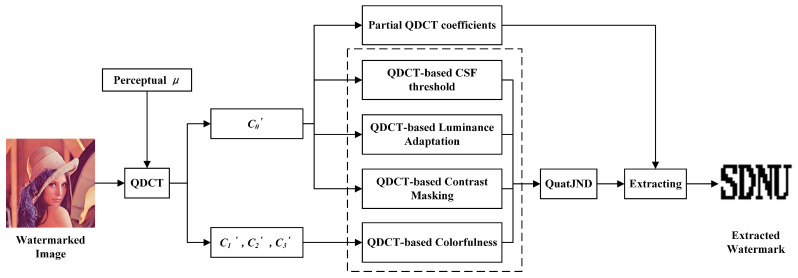
The flowchart of the proposed watermark extracting scheme.

The extracting algorithm is an inverse procedure of the embedding algorithm. Figure 4 illustrates the extracting steps of the watermarking scheme. And the procedures of the watermark extracting are shown as follows:

Step 1: For a watermarked image, it is first and divided into non-overlapped blocks of 8×8 size, and each block is converted to the quaternion representation by Equation (4).

Step 2: Apply QDCT which used the perceptual unit pure quaternion μ to each block, and the QDCT spectrum coefficients are obtained by Equation (11).

Step 3: Estimate the QuatJND factors including the spatial CSF effect, luminance adaptation and contrast masking in ${C_{0}}^{\prime }$ quaternion domain by Equations (20), (25) and (29), respectively.

Step 4: Extract colorfulness feature from ${C_{1}}^{\prime }$, ${C_{2}}^{\prime }$ and ${C_{3}}^{\prime }$ by Equation (39). Quantize and calculate the colorfulness masking of each 8×8 block for QuatJND profile by Equation (42).

Step 5: Final QuatJND value of each block combined with colorfulness masking is determined by Equation (19). The proposed QuatJND value can be served as the perceptual redundancy vector $S^{\prime }$.

Step 6: The ${C_{0}}^{\prime }$ coefficients from the fourth to tenth except the fifth after zigzag scan are selected to form a host vector $X^{\prime }$. The host vector $X^{\prime }$ and the perceptual redundancy vector $S^{\prime }$ are used to obtain the transformed vector $Y^{\prime }$ and the adaptive quantization step $\Delta^{\prime }$.

Step 7: The watermark can be detected according to the minimum distance detector as follows

(47) $$w^{\prime }=\underset{b\in \{0,1\}}{\arg\min}\;\left|Y^{\prime }-Q\left(Y^{\prime },b,\Delta^{\prime },d_{m}\right)\right|$$

Step 8: The final watermark image is obtained by the inverse Arnold transform.

## 4. Experimental Results and Comparisons

In this section, we show and discuss the experimental results. To prove the effectiveness and robustness performance of our proposed scheme, we perform experiments using the original code in MATLAB (MathWorks, Natick, MA, USA) R2019a on a 64-bit Windows 10 operating system at 16 GB memory, 3.40 GHz frequency of Intel (R) Core (TM) i7-6700 CPU (Intel, Santa Clara, CA, USA).

### 4.1. Performance Metrics

In the experiments, two objective criteria include Peak Signal to Noise Ratio (PSNR) and Quaternion Structural Similarity Index (QSSIM) have been considered to measure the fidelity. The Bit Error Rate (BER) is computed to evaluate the robustness of algorithms.

(1) Peak Signal to Noise Ratio (PSNR)

PSNR provides an objective standard for measuring image distortion or noise level. In this experiment, we use PSNR to evaluate the quality between the embedded image and original image, which means it is used to evaluate the invisibility of the embedded watermark. The evaluation result is expressed in dB (decibel). The larger the PSNR value between the two images, the better the invisibility of the watermarking scheme. Considering the host color image *I* of size M×N and its watermarked version $I^{\prime }$, the PSNR is defined as

(48) $$\begin{array}{cc} \; & PSNR=10\mathrm{lg}[\frac{255^{2}}{\frac{1}{3MN}{\sum_{x=1}^{M}\sum_{y=1}^{N}\sum_{\theta \in \{R,G,B\}}\left(I_{\theta }\left(x,y\right)-{I_{\theta }}^{\prime }\left(x,y\right)\right)}^{2}}] \end{array}$$

(2) Quaternion Structural Similarity Index (QSSIM)

Kolaman et al. [31] developed a visual quality matrix that will be able to better evaluate the quality of color images, which is named quaternion SSIM (QSSIM). The value of QSSIM ranges is [0, 1]. And the closer the QSSIM value is to 1, the better the image’s visual quality effect. The QSSIM is defined by Equation (49), which is composed to be the same as SSIM but with quaternion subparts.

(49) $$QSSIM=\left|\left(\frac{2\mu_{qI}\cdot \mu_{qI^{\prime }}}{\mu_{{}_{qI}}^{2}+\mu_{{}_{qI^{\prime }}}^{2}}\right)\left(\frac{\sigma_{qI,qI^{\prime }}}{\sigma_{{}_{qI}}^{2}+\sigma_{{}_{qI^{\prime }}}^{2}}\right)\right|$$

where,

qI, $qI^{\prime }$ are the quaternion representation (QR) of image *I* and its watermarked version $I^{\prime }$ respectively;

$\mu_{qI}$, $\mu_{qI^{\prime }}$ are the mean of image *I* and its watermarked version $I^{\prime }$ respectively;

$\sigma_{{}_{qI}}^{2}$, $\sigma_{{}_{qI^{\prime }}}^{2}$ are the variance of image *I* and its watermarked version $I^{\prime }$ respectively;

$\sigma_{qI,qI^{\prime }}$ is the covariance of image *I* and its watermarked version $I^{\prime }$.

(3) Bit Error Rate (BER)

The Bit Error Rate was here utilized to evaluate the quality of the extracted binary watermark image $w^{\prime }$ compared to its original version *w*, both of $M_{w}\times N_{w}$ pixels. The BER between $w^{\prime }$ and *w* is given by

(50) $$BER=\frac{\sum_{x=1}^{M_{w}}\sum_{y=1}^{N_{w}}w^{\prime }\left(x,y\right)⊕w\left(x,y\right)}{M_{w}\times N_{w}}$$

### 4.2. Imperceptibility

**Figure 5:**
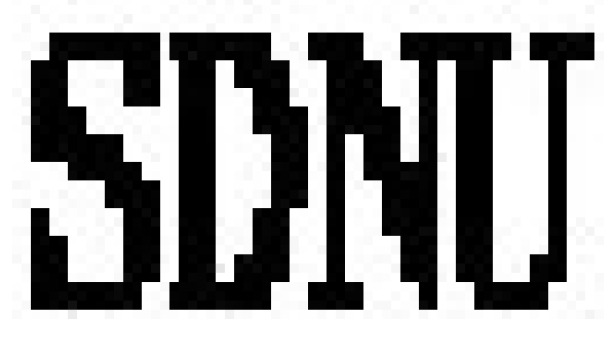
Watermark image.

**Figure 6:**
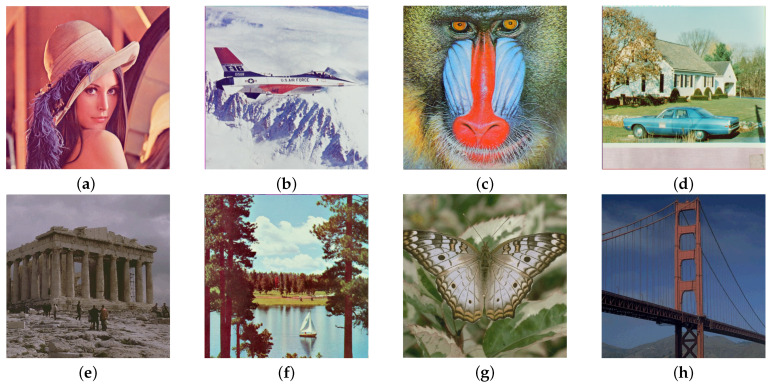
Original cover images. (**a**) Lena, (**b**) Avion, (**c**) Baboon, (**d**) House, (**e**) Athens, (**f**) Sailboat, (**g**) Butrfly, (**h**) Goldgate.

To verify the performance of the proposed color image watermarking algorithm, 109 color images available from the Computer Vision Group at the University of Granada (http://decsai.ugr.es/cvg/dbimagenes/, accessed on 21 September 2020) were considered. A binary watermark “SDNU” of length 4096 bits (64×64) is embedded into the original cover images as shown in Figure 5. Eight standard images ‘Lena’, ‘Avion’, ‘Baboon’, ‘House’, ‘Athens’, ‘Sailboat’, ‘Butrfly’ and ‘Goldgate’, were used as testing images. The size of the eight testing images are 512×512 shown in Figure 6.

**Figure 7:**
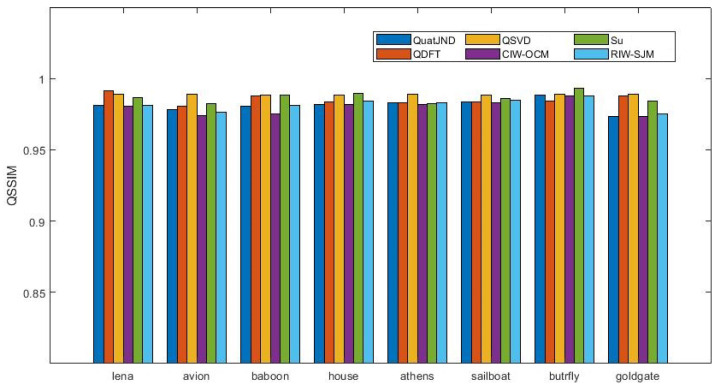
QSSIM comparison of different models (QDFT [16], QSVD [32], CIW-OCM [10], Su [33], RIW-SJM [11]) with PSNR = 42 dB.

For evaluating the invisibility of the embedded watermark, we embed the watermark in Figure 5 in the host images in Figure 6a–h, respectively. And the proposed scheme was compared with the popular watermarking schemes, referring to QDFT [16], QSVD [32], Wang et al. [10] proposed color image watermarking based on orientation diversity and color complexity (CIW-OCM), Wang et al. [11] proposed robust image watermarking via perceptual structural regularity-based JND model (RIW-SJM), and Su [33]. First of all, a good watermarking scheme must show a satisfying invisibility. Figure 7 gives the visual quality fraction of the watermarking images. The tested images in Figure 7 are first restrained with the same PSNR = 42 dB and we ensure this by modifying the embedded intensity factor. The QSSIM values are compared, the higher the QSSIM value, the more complete the details and structure of the image preserved. The average QSSIM values of different algorithms are 0.9850, 0.9886, 0.9794, 0.9814 and 0.9864, respectively, and the proposed QSSIM value is 0.9810. Although the results for the proposed image watermarking scheme are not the best compared with other watermarking schemes, the QSSIM values are almost similar to other schemes on average. With the same PSNR guaranteed, the QSSIM of our scheme is comparable to other schemes. This is because in order to achieve a balance between imperceptibility and robustness, our scheme satisfies the imperceptibility while calculating the redundancy of the image more accurately, making the modification of the image larger. Thus the algorithm in this paper can obtain better robustness while satisfying the imperceptibility, while the tests of robustness in Section 4.3 below also demonstrate this.

**Figure 8:**
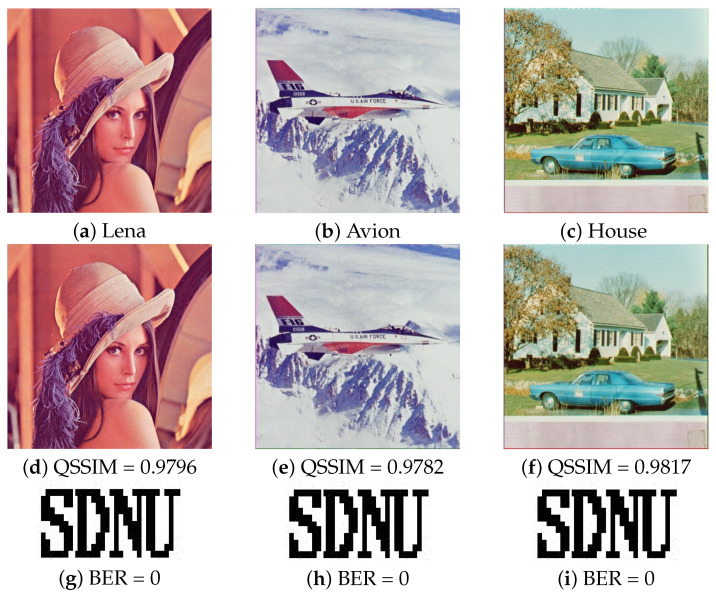
Experimental results without attack (**a**–**c**) Cover image; (**d**–**f**) watermarked image; (**g**–**i**) extracted watermark from (**d**–**f**).

To prove that the proposed image watermarking scheme can produce a high watermark quality and the watermark can be extracted correctly without attack. The test images were watermarked with the uniform fidelity, a fixed Peak Signal to Noise Ratio (PSNR) of 42 dB. The bit error rate (BER) was computed to make the objective performance evaluation. Figure 8 shows the cover images, watermarked images, and extracted watermarks. Intuitively, it is noticeable that the proposed method can provide a good visual quality of the extracted watermark image.

### 4.3. Robustness

#### 4.3.1. Evaluation of Different Unit Pure Quaternions

In order to prove that the perceptual unit pure quaternion in Section 3.1 can produce a better watermark quality, we compare the robustness results with different types of pure unit quaternion such as $\mu_{1}=\left(-2j+8k\right)/\sqrt{68}$ [13], $\mu_{2}=\left(j-k\right)/\sqrt{2}$ [34], and $\mu_{3}=\left(i+j+k\right)/\sqrt{3}$ [20]. It should be noticed that $\mu_{3}$ is the most common unit quaternion used in the quaternion based on image processing literature. Table 2 shows the performance for different μ. From the results, the perceptual unit quaternion has lower BER in JPEG compression. This shows the advantages of QDCT transform itself which is compatible with the JPEG compression standard. Although for the perceptual unit quaternion, the performance is not the best as others under Gaussian noise and Filtering, it also has low BER and shows good robustness. In total, the perceptual pure unit quaternion μ has better performance against common signal attacks, especially in JPEG attacks.

#### 4.3.2. Evaluation of Different JND Models within QDCT Watermarking Algorithm

This experiment is used to compare the performance of different JND models used within the proposed QDCT watermarking algorithm. To verify robustness performance of our proposed QuatJND model guided watermarking scheme, the proposed scheme is compared with different JND models, referring to Watson’s model [4], Kim’s model [6] and Zhang’s model [7].

**Figure 9:**
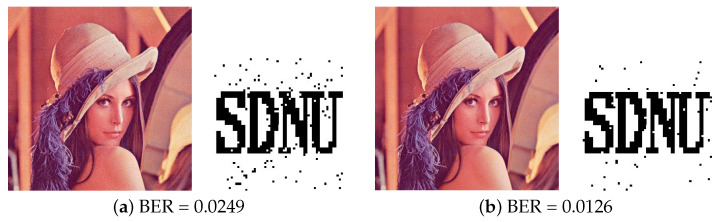

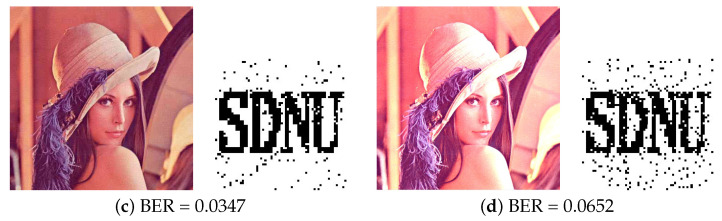
Results under different types of attacks and recovered watermark from Lena image. (**a**) Gaussian Noise (var = 0.0015) (**b**) Salt and Pepper noise (density = 0.0015) (**c**) JPEG compression (Q = 30) (**d**) Amplitude Scaling 1.5.

**Figure 10:**
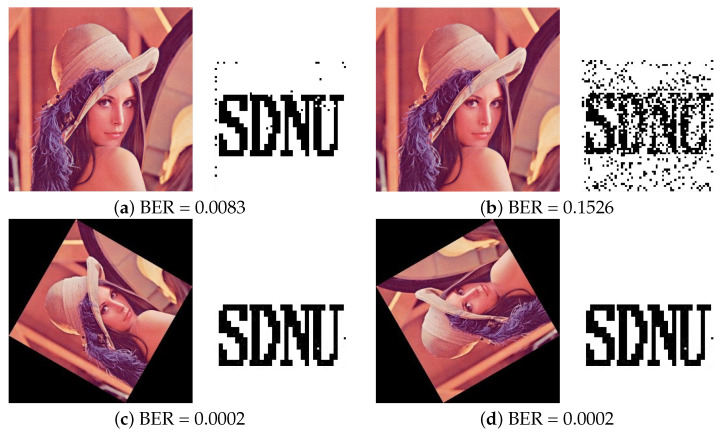
Results under different types of attacks and recovered watermark from Lena image. (**a**) Gaussian filtering (3,3) (**b**) Median filtering (3,3) (**c**) Image rotation (angle = 60) (**d**) Image rotation (angle = 120).

In this experiment, we recomputed the features of Watson’s model, Kim’s model and Zhang’s model in the quaternion DCT domain, respectively. For example, in Kim’s model, we used the $C_{0}$ coefficients to calculate the base threshold, luminance adaptation, and contrast masking in the quaternion domain. The tested images are first embedded watermark and restrained with the same PSNR = 42 dB, and the average BER values are compared. As shown in Table 3, compared with the other JND models, the proposed model always has the lowest BER for different noise intensities. This indicates that the proposed model performed much better than others. As for JPEG compression, different performance emerges in the four JND models within the watermarking algorithms shown about JPEG compression attacks. The average BER of Watson’s, Kim’s, Zhang’s and QuatJND model are 0.0828, 0.1144, 0.0775, and 0.0331 when JPEG compression quality is 30, respectively. And from Figure 9c, the extracted watermark can be clearly identified when JPEG compression quality is 30. When the Median filtering and Gaussian filtering are used to attack the watermarked image. For filtering with median filter (3,3), the BER value of the proposed model is 4.5% higher than the Kim’s model, but in Figure 10b, the extracted watermark can also be correctly recognized. In summary, our proposed QuatJND model performs excellently in quaternion domain.

#### 4.3.3. Evaluation of Watermarking Algorithms in Different Domains

This experiment is used to compare the performance of different watermarking algorithms in DCT domain and spatial domain. To verify the effectiveness of quaternion DCT and the advantage of the quaternion, the proposed scheme is compared with CIW-OCM [10], RIW-SJM [11]) and Su [33].

(1) Under common attacks

During the image transmission, the watermarked image is attacked easily and inevitably by some common attacks such as Gaussian noise, Salt and Pepper noise, JPEG compression and Amplitude scaling. Table 4 lists the average robustness results for eight test images using the different watermarking schemes under various attacks, such as Gaussian Noise with zero mean, variances 0.0003, 0.0008 and 0.0012; Salt and Pepper noise with different densities 0.004, 0.008 and 0.015; JPEG compression with quality factors 30, 50, and 80; Amplitude scaling with factors 0.3, 1.2 and 1.5.

First, it is obvious that our proposed scheme can get a minimum bit error rate compared with other schemes after Gaussian noise and Salt and Pepper noise attack from Table 4. As shown in the robustness results, the proposed scheme has a lower average BER value than CIW-OCM [10] about 0.3% at least. And with the density of Salt and Pepper noise increased, Su [33] shows lower BER than ours when density is 0.015. For traditional JPEG compression attacks, our proposed scheme has similar results when JPEG compression quality is greater than 50, which is 0.1–0.4% lower than the CIW-OCM [10]. In general, the proposed model has the best performance against JPEG compression attacks on average. Finally, while the watermarked image is distorted by Amplitude Scaling attack, although the performance of the proposed model is not the best, the results are almost similar to other schemes on average. And from Figure 9d, the extracted watermark can be clearly identified when the Amplitude Scaling is 1.5, which can satisfy the robustness of watermarking scheme against Amplitude Scaling attacks.

(2) Under filtering attacks

Filtering attacks such as Median filtering and Gaussian filtering are usually used to attack the watermarked image. And the visual perception of the extracted watermark can be destroyed by these attacks. The performance of watermarking model resists the Filter attacks needs to be considered. Table 5 and Figure 10a,b present the comparison results of filtering. For filtering with Median filtering (3,3), the BER value of the proposed model is 1% lower than the model of RIW-SJM [11]. And for Gaussian filtering, the proposed model has the lowest BER values than the rest of models, which can ensure that the extracted watermark image has a higher recognition.

(3) Under cropping attacks

**Figure 11:**
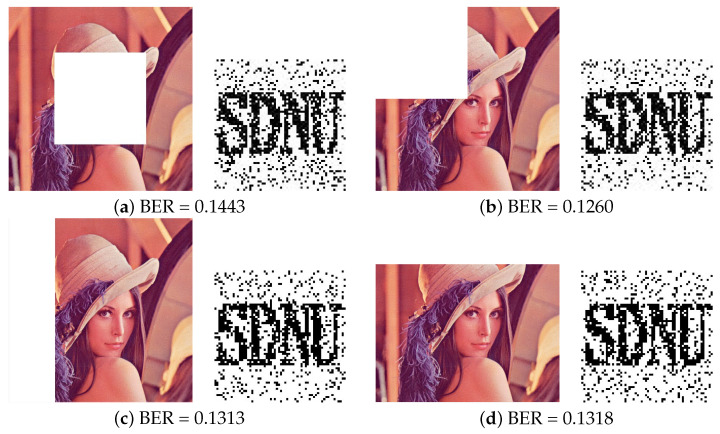
Results under different types of attacks and recovered watermark from Lena image. (**a**) Central cropping 1/4 (**b**) Left upper cropping 1/4 (**c**) Row cropping 1/4 (**d**) Column cropping 1/4.

In practice, the watermarked image will also be contaminated by other attacks such as Cropping and geometric attacks. Here, in this experiment, image rotation has been considered as a geometric attack, which results in the change of image pixel value and image size. We firstly compared the robustness results after Cropping attacks in Table 6 and Figure 11. The watermarking image is affected by Central cropping (1/8 of image), Left upper cropping (1/8 of image), Row cropping (1/8 of image) and Column cropping (1/8 of image). From the results of Table 6, the proposed model gets the lowest BER value than other algorithms which means that the proposed method provides a good visual quality of the extracted watermark image after different types of cropping attacks.

(4) Under rotation attacks

To verify that the proposed image watermarking scheme can be robust to geometric attacks, we test the robustness of the proposed algorithm after image Rotation. In this experiment, one watermarking image is first carried out a forward image Rotation transformation, and is then corrected by an inverse image Rotation transformation. More clearly, the watermarking image rotates clockwise 30, 60, 90, 120, and then rotates counter-clockwise to extract the watermarking.

The robustness of image rotation is listed in Table 7 and Figure 10c,d. The results show that our proposed method has the lowest BER value than other methods. For rotation, the value of BER obtained by our method does not exceed 0.2% when the rotation angle is 30, 60, 90, 120, which demonstrates our method can get a significant robustness performance for image rotation.

#### 4.3.4. Evaluation of Different Quaternion Watermarking Schemes

This experiment is used to compare the performance of different quaternion watermarking algorithms. To verify the robustness of the proposed scheme in quaternion DCT domain, the proposed scheme is compared with QDFT [16] and QSVD [32].

Table 8 shows the BER values of watermarked images attacked by Gaussian Noise (GN), JPEG compression attacks, Salt and Pepper noise (SPN), Median filtering (MF) Gaussian filtering (GF) and Amplitude Scaling (AS). As shown in the robustness results, for traditional JPEG compression attack, both QSVD [32] and QDFT [16] have a poorer performance than the proposed scheme, the reason may be that the proposed scheme enhances the performance to resist JPEG attack by using QDCT domain. When the watermarked image is distorted by Amplitude Scaling attack, the performance of the proposed scheme is better than that of other schemes except QSVD [32]. In QSVD [32], they inserted the watermark through moderating the coefficients $f_{1,1}$ and $f_{2,1}$ of the quaternion elements in U matrix, the Amplitude Scaling attack leads to the minimum effect on the relative relationship between $f_{1,1}$ and $f_{2,1}$. Therefore, QSVD [32] shows superior performance to Amplitude Scaling attack.

Table 9 demonstrates the comparision of the average BER values between our scheme and other methods for different image attacks with fixed image quality, QSSIM = 0.9820. Although the QDFT [16] has better robustness to against the process of adding Gaussian noise and Salt and Pepper noise, the scheme has a poorer performance in JPEG compression. For JPEG compression, it can be seen from Table 9 that our method has a lower BER than other watermarking algorithms when the JPEG with QF is 30 and 50. In addition, the robustness performance of our method is obviously better than others under combined image attacks that performed JPEG compression firstly, followed by the Gaussian noise and Salt and Pepper noise. Above all, the watermarking framework based on the QuatJND model in QDCT domain has better robustness performance than other methods in most cases.

#### 4.3.5. Evaluation of Combined Attacks

**Figure 12:**
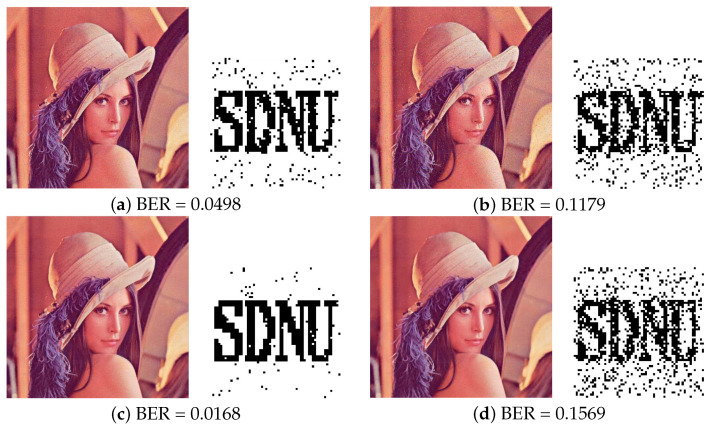
Results under different types of attacks and recovered watermark from Lena image. (**a**) JPEG 30 + Gaussian Noise (var = 0.0015) (**b**) JPEG 30 + Salt and Pepper noise (density = 0.0015) (**c**) JPEG 30 + Gaussian filtering (3,3) (**d**) JPEG 30 + Median filtering (3,3).

**Figure 13:**
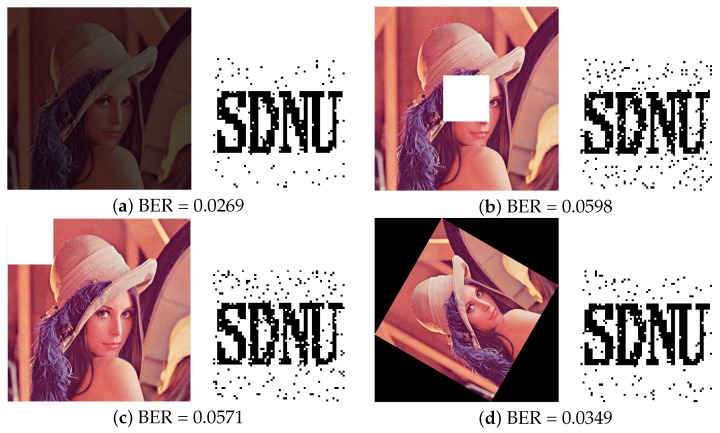
Results under different types of attacks and recovered watermark from Lena image. (**a**) Gaussian Noise (var = 0.0015) + Amplitude Scaling 0.3 (**b**) Gaussian Noise (var = 0.0015) + Central cropping 1/16 (**c**) Gaussian Noise (var = 0.0015) + Left upper cropping 1/16 (**d**) Gaussian Noise (var = 0.0015) + Image rotation (angle = 60).

Table 3, Table 4, Table 5, Table 6, Table 7, Table 8 and Table 9 list the robustness performance after single image attack. However, in the actual digital signal transmission process, the watermarked image will be destroyed by multiple attacks simultaneously. Here, we further compared the robustness results after various combined attacks by common image processing in Figure 12 and Figure 13. Figure 12 shows the BER after passing JPEG compression processing, followed by common Gaussian noise, Salt and Pepper noise, Gaussian filtering and Median filtering attacks.

Figure 13 shows the BER after passing Gaussian noise, followed by Amplitude Scaling, Cropping and image Rotation. From the results of Figure 12 and Figure 13, the human eye still can recognize the extracted watermark information after different combined attacks. In summary, our method shows well robustness performance after combinatorial image attacks, which means that our method can achieve good image copyright protection in practical applications.

On the whole, some exist quaternion watermarking algorithms such as QSVD [32] and QDFT [16], some DCT domain watermarking algorithms such as CIW-OCM [10] and RIW-SJM [11], and Su [33] which is an improved watermarking algorithm based on Schur decomposition, although these algorithms show better invisibility for watermarked images from the results of Figure 7, these algorithms have poorer robustness under some attacks. They can’t achieve a good tradeoff between invisibility and robustness. As for CIW-OCM [10] and RIW-SJM [11], although the algorithm achieves a good tradeoff by using JND models, the algorithm neglects the correlation between the three color components. The proposed model exploits the correlation for three color channels and uses a QuatJND model to obtain the optimum quantization step, and the results show our scheme has better robust performance than others.

## 5. Conclusions

In this paper, we proposed a robust quaternion JND model for color image watermarking (QuatJND). Firstly, we obtained the quaternion DCT coefficients by the perceptual unit pure quaternion. And then QuatJND model is calculated by using the quaternion DCT coefficients. The color information is also considered. A logarithmic STDM scheme is further proposed based on the QuatJND. Our scheme is evaluated under different types of attacks such as Gaussian noise, JPEG compression, Gaussian filter, Median filter and geometrical attacks like image rotation, cropping. The proposed technique also provides robustness results under combined attacks. Experimental results show that our scheme provides better robust performance than existing techniques. Color is a very important content in images. We can further use the color information in the images to enhance the accuracy of the QuatJND model. For example, the cross-masking effect of luminance and color components can be further analyzed in order to enhance the imperceptibility of watermarked images in future research. Meanwhile, deep learning methods can extract image features more effectively and build a more accurate JND model to make its robust performance under various attacks more effective.

## Figures and Tables

**Table 1:** The relevant abbreviations and symbols used in this paper.

Symbols	Meaning	Symbols	Meaning
JND	Just noticeable difference	$\omega_{m,n}$	Cycle per degree
QuatJND	Quaternion JND model	$\varphi_{m,n}$	The direction angle
PSNR	Peak Signal to Noise Ratio	$J_{q\_d}$, $J_{q\_v}$	The oblique effect
QSSIM	Quaternion Structural Similarity Index	$\mu_{la}$	Average intensity
QDCT	Quaternion Discrete Cosine Transform	$E_{d}$	Max direction feature
QDFT	Quaternion Discrete Fourier Transform	$\mu_{cm}$	Contrast masking effect
QSVD	Quaternion Singular Value Decomposition	$C_{p}$	Pattern complexity
*q*	A quaternion	$C_{l}$	Luminance contrast
*a*,*b*,*c*,*d*	Four real numbers of a quaternion	$K_{1_{\left(R-G\right)}}$	Red-Green color space
*i*,*j*,*k*	Three imaginary numbers of a quaternion	$K_{2_{\left(Y-B\right)}}$	Yellow-Blue color space
μ	Unit pure quaternion	$\mu_{K_{1}}^{}$	Mean value
C(p,s)	QDCT coefficients	$\sigma_{K_{1}}^{2}$	Variance value
$C_{0}\left(p,s\right)$, $C_{1}\left(p,s\right)$, $C_{2}\left(p,s\right),C_{3}\left(p,s\right)$	Four parts of C(p,s)	*v*	Random vector
$\bar{f}\left(x,y\right)$	Inverse QDCT coefficients	*z*	Secret key
$\bar{f_{0}}\left(x,y\right)$, $\bar{f_{1}}\left(x,y\right)$, $\bar{f_{2}}\left(x,y\right)$, $\bar{f_{3}}\left(x,y\right)$	Four parts of $\bar{f}\left(x,y\right)$	*S*	Slack vector
$C_{0}\left(0,0\right)$	Quaternion DC coefficients of $C_{0}\left(p,s\right)$	*X*	Host vector
$C_{0}\left(0,1\right)$, $C_{0}\left(1,0\right)$, $C_{0}\left(1,1\right)$	Quaternion AC coefficients of $C_{0}\left(p,s\right)$	*Y*	Transformed vector
$M_{q\_LA}$	Luminance adaptation effect	$d_{m}$	Dither signal
$M_{q\_COL}$	Colorfulness masking	*w*	Watermark bit
$J_{q\_base}$	The base CSF threshold	Δ	Quantization step
$M_{q\_CM}$	Contrast masking	$Y_{w}$	Quantization vector
$Q_{c}$	Colorfulness value	$w^{\prime }$	Extracted watermark
RIW-SJM	Wang et al. [11]		
CIW-OCM	Wang et al. [10]		

**Table 2:** BER comparison results of ‘Lena’ with PSNR = 42 dB.

Attack	$\mu_{1}$	$\mu_{2}$	$\mu_{3}$	Perceptual μ
JPEG 30	0.0983	0.0893	0.0813	**0.0352**
JPEG 50	0.0346	0.0324	0.0115	**0.0008**
JPEG 80	0.0155	0.0195	0.0034	**0.0000**
Gaussian noise 0.0008	0.0042	0.1423	**0.0039**	0.0042
Salt and Pepper noise 0.008	**0.0498**	0.1531	0.0579	0.0617
Amplitude Scaling 0.5	**0.0000**	0.0049	**0.0000**	**0.0000**
Median filtering (3,3)	0.1458	0.1528	0.1555	**0.1408**
Gaussian filtering (3,3)	**0.0063**	0.0381	0.0110	0.0083

**Table 3:** Average BER with different JND models.

Attack	Watson Model [4]	Kim Model [6]	Zhang Model [7]	QuatJND Model
Gaussian noise 0.0003	0.0029	0.0189	0.0085	**0.0005**
Gaussian noise 0.0008	0.0249	0.0356	0.0195	**0.0065**
Salt and Pepper noise 0.004	0.0466	0.0572	0.0454	**0.0289**
Salt and Pepper noise 0.008	0.0821	0.0947	0.0825	**0.0562**
JPEG 30	0.0828	0.1144	0.0775	**0.0331**
JPEG 50	0.0167	0.0253	0.0127	**0.0018**
Gaussian filtering (3,3)	0.0344	0.0248	0.0224	**0.0136**
Median filtering (3,3)	0.1861	**0.1467**	0.1551	0.1917
Rotation 30°	0.0030	0.0183	0.0465	**0.0014**
Rotation 60°	0.0036	0.0185	0.0143	**0.0016**

**Table 4:** Average BER comparison results with PSNR = 42 dB.

Attack	CIW-OCM [10]	RIW-SJM [11]	Su [33]	Proposed
Gaussian noise 0.0003	0.0008	0.0043	0.0052	**0.0005**
Gaussian noise 0.0008	0.0135	0.0343	0.0172	**0.0065**
Gaussian noise 0.0012	0.0298	0.0668	0.0312	**0.0172**
Salt and Pepper noise 0.004	0.0620	0.0363	0.0302	**0.0289**
Salt and Pepper noise 0.008	0.1157	0.0694	0.0568	**0.0562**
Salt and Pepper noise 0.015	0.1394	0.2039	**0.0921**	0.1089
JPEG 30	0.0589	0.1265	0.1544	**0.0331**
JPEG 80	0.0002	0.0008	0.0326	**0.0001**
Amplitude Scaling 0.3	0.0037	0.0178	0.1311	**0.0001**
Amplitude Scaling 1.2	**0.0207**	0.0204	0.0684	0.0216
Amplitude Scaling 1.5	0.1370	0.1702	0.1241	**0.1289**

**Table 5:** Average BER of filtering attacks with PSNR = 42 dB.

Attack	CIW-OCM [10]	RIW-SJM [11]	Su [33]	Proposed
Median filtering (3,3)	0.2062	0.2016	0.2335	**0.1917**
Gaussian filtering (3,3)	0.0183	0.0258	0.0142	**0.0136**

**Table 6:** Average BER of cropping attacks with PSNR = 42 dB.

Attack	CIW-OCM [10]	RIW-SJM [11]	Su [33]	Proposed
Central cropping 1/8	0.0097	0.0099	0.0127	**0.0091**
Left upper cropping 1/8	0.0162	0.0579	0.0456	**0.0129**
Row cropping 1/8	0.0707	0.0730	0.0831	**0.0674**
Column cropping 1/8	0.0703	0.0786	0.0873	**0.0646**

**Table 7:** Average BER of Rotation attacks with PSNR = 42 dB.

Attack	CIW-OCM [10]	RIW-SJM [11]	Su [33]	Proposed
Rotation 30	0.0034	0.0027	0.0039	**0.0014**
Rotation 60	0.0039	0.0037	0.0035	**0.0016**
Rotation 90	**0.0000**	**0.0000**	**0.0000**	**0.0000**
Rotation 120	0.0034	0.0027	0.0039	**0.0014**

**Table 8:** BER of different attacks with PSNR = 42 dB.

Attack	Lena			House		
	QDFT [16]	QSVD [32]	Proposed	QDFT [16]	QSVD [32]	Proposed
GN (0.0008)	**0.0007**	0.0135	0.0059	0.0051	0.0562	**0.0042**
GN (0.0012)	**0.0095**	0.0225	0.0149	0.0181	0.0928	**0.0171**
SPN (0.004)	0.0405	**0.0271**	0.0291	0.0359	0.0571	**0.0332**
SPN (0.008)	0.0906	**0.0525**	0.0640	0.0842	0.1060	**0.0524**
JPEG (30)	0.3043	0.3400	**0.0347**	0.3355	0.3558	**0.0308**
JPEG (50)	0.1270	0.2076	**0.0007**	0.2250	0.2787	**0.0022**
GF (3,3)	0.0095	0.0432	**0.0083**	0.0225	0.0945	**0.0149**
MF (3,3)	0.1775	0.4724	**0.1526**	0.2100	0.4846	**0.2063**
AS (0.3)	0.0454	**0.0001**	**0.0001**	0.1240	**0.0002**	0.0004
AS (1.2)	0.0249	**0.0004**	0.0029	0.0862	**0.0007**	0.0461

**Table 9:** Average BER of different attacks with QSSIM = 0.9820.

Attack	CIW-OCM [10]	RIW-SJM [11]	Su [33]	QDFT [16]	QSVD [32]	Proposed
GN (0.0008)	0.0157	0.0108	0.0100	**0.0003**	0.0207	0.0083
GN (0.0012)	0.0337	0.0249	0.0183	**0.0006**	0.0367	0.0203
SPN (0.004)	0.0380	0.0330	0.0242	**0.0238**	0.0376	0.0248
SPN (0.008)	0.0735	0.0648	0.0441	**0.0440**	0.0513	0.0554
JPEG (30)	0.0708	0.0589	0.0983	0.2739	0.2866	**0.0401**
JPEG (50)	0.0047	0.0034	0.0660	0.1278	0.2063	**0.0029**
GF (3,3)	0.0160	0.0116	**0.0048**	0.0069	0.0435	0.0112
MF (3,3)	0.1949	0.1845	0.1983	0.2628	0.4626	**0.1807**
AS (0.3)	0.0037	0.0006	0.1451	0.0476	**0.0001**	**0.0001**
AS (1.2)	0.0211	0.0210	0.0733	0.0177	**0.0006**	0.0170
JPEG (50) + GN (0.0008)	0.0415	0.0324	0.1195	0.1556	0.2168	**0.0254**
JPEG (50) + GN (0.0012)	0.0596	0.0498	0.1480	0.1734	0.2301	**0.0394**
JPEG (50) + SPN (0.004)	0.0516	0.0444	0.1435	0.1499	0.2122	**0.0367**
JPEG (50) + SPN (0.008)	0.0905	0.0764	0.1554	0.1683	0.2208	**0.0672**

## Data Availability

Not applicable.

## References

[1] Su Q., Chen B. (2018). Robust color image watermarking technique in the spatial domain. Soft Comput..

[2] Tsui T.K., Zhang X.P., Androutsos D. (2008). Color image watermarking using multidimensional Fourier transforms. IEEE Trans. Inf. Forensics Secur..

[3] Thongkor K., Amornraksa T. Digital image watermarking with partial embedding on blue color component. In Proceedings of Asia-Pacific Signal and Information Processing Association Annual Summit and Conference 2014 (APSIPA).

[4] Watson A.B. DCT quantization matrices visually optimized for individual images. Proceedings of the Human Vision, Visual Processing, and Digital Display IV.

[5] Ma L., Yu D., Wei G., Tian J., Lu H. (2010). Adaptive spread-transform dither modulation using a new perceptual model for color image watermarking. IEICE Trans. Inf. Syst..

[6] Bae S.H., Kim M.A. (2013). A novel DCT-based JND model for luminance adaptation effect in DCT frequency. IEEE Signal Process. Lett..

[7] Zhang X.H., Lin W.S., Xue P. (2005). Improved estimation for just-noticeable visual distortion. Signal Process..

[8] Wan W., Liu J., Sun J., Gao D. (2016). Improved logarithmic spread transform dither modulation using a robust perceptual model. Multimed. Tools Appl..

[9] Wan W., Wang J., Li J., Meng L., Sun J., Zhang H., Liu J. (2020). Pattern complexity-based JND estimation for quantization watermarking. Pattern Recognit. Lett..

[10] Wang J., Wan W., Li X., Sun J., Zhang H. (2020). Color image watermarking based on orientation diversity and color complexity. Expert Syst. Appl..

[11] Wang C., Xu M., Wan W., Wang J., Meng L., Li J., Sun J. (2019). Robust image watermarking via perceptual structural regularity-based JND model. Ksii Trans. Internet Inf. Syst..

[12] Tsui T.K., Zhang X.P., Androutsos D. Quaternion image watermarking using the spatio-chromatic fourier coefficients analysis. Proceedings of the 14th ACM International Conference on Multimedia.

[13] Bas P., Le Bihan N., Chassery J.M. Color image watermarking using quaternion Fourier transform. Proceedings of the 2003 IEEE International Conference on Acoustics, Speech, and Signal Processing.

[14] Ma X., Xu Y., Song L., Yang X., Burkhardt H. Color image watermarking using local quaternion Fourier spectral analysis. Proceedings of the 2008 IEEE International Conference on Multimedia and Expo.

[15] Jiang S.H., Zhang J.Q., Hu B. (2009). Content based image watermarking algorithm in hypercomplex frequency domain. Syst. Eng. Electron..

[16] Chen B., Coatrieux G., Chen G., Sun X., Coatrieux J.L., Shu H. (2014). Full 4-D quaternion discrete Fourier transform based watermarking for color images. Digit. Signal Process..

[17] Yanshan L. A new color image blind watermarking algorithm based on quaternion. Proceedings of the IEEE 10th International Conference on Signal Processing Proceedings.

[18] Liu F., Ma L.H., Liu C., Lu Z.M. (2018). Optimal blind watermarking for color images based on the U matrix of quaternion singular value decomposition. Multimed. Tools Appl..

[19] Li J., Lin Q., Yu C., Ren X., Li P. (2018). A QDCT and SVD-based color image watermarking scheme using an optimized encrypted binary computer-generated hologram. Soft Comput..

[20] Feng W., Hu B. Quaternion discrete cosine transform and its application in color template matching. Proceedings of the 2008 Congress on Image and Signal Processing.

[21] Pei S.C., Ding J.J., Chang J.H. (2001). Efficient implementation of quaternion Fourier transform, convolution, and correlation by 2-D complex FFT. IEEE Trans. Signal Process..

[22] Zhu S.Y., He Z.Y., Chen C., Liu S.C., Zhou J., Guo Y., Zeng B. (2019). High-quality color image compression by quantization crossing color spaces. IEEE Trans. Circuits Syst. Video Technol..

[23] Koju R., Joshi S.R. (2014). Comparative analysis of color image watermarking technique in RGB, YUV, and YCbCr color channels. Nepal J. Sci. Technol..

[24] Roy A., Maiti A.K., Ghosh K. (2018). An HVS inspired robust non-blind watermarking scheme in YCbCr Color Space. Int. J. Image Graph..

[25] Wan W., Wang J., Li J., Sun J., Zhang H., Liu J. (2020). Hybrid JND model-guided watermarking method for screen content images. Multimed. Tools Appl..

[26] Wang J., Wan W. (2020). A novel attention-guided JND Model for improving robust image watermarking. Multimed. Tools Appl..

[27] Muthuswamy K., Rajan D. (2013). Salient motion detection in compressed domain. IEEE Signal Process. Lett..

[28] Hasler D., Suesstrunk S.E. Measuring colorfulness in natural images. In Proceedings of Human Vision and Electronic Imaging VIII.

[29] Panetta K., Gao C., Agaian S. (2013). No reference color image contrast and quality measures. IEEE Trans. Consum. Electron..

[30] Ponomarenko N., Lukin V., Zelensky A., Egiazarian K., Carli M., Battisti F. (2009). TID2008—A Database for Evaluation of Full-Reference Visual Quality Assessment Metrics. Adv. Mod. Radioelectron..

[31] Kolaman A., Yadid-Pecht O. (2011). Quaternion Structural Similarity: A New Quality Index for Color Images. IEEE Trans. Image Process..

[32] Liu F., Feng H., Lu C. (2015). Blind watermarking scheme based on U matrix through QSVD transformation. Int. J. Secur. Its Appl..

[33] Su Q., Zhang X., Wang G. (2020). An improved watermarking algorithm for color image using Schur decomposition. Soft Comput..

[34] Ell T.A., Sangwine S.J. Decomposition of 2D hypercomplex Fourier transforms into pairs of complex Fourier transforms. In Proceeding of the 10th European Signal Processing Conference.

